# Percutaneous muscle biopsy‐induced tissue injury causes local endoplasmic reticulum stress

**DOI:** 10.14814/phy2.13679

**Published:** 2018-04-24

**Authors:** Jun Yoshino, Paloma Almeda‐Valdes, Anna C. Moseley, Bettina Mittendorfer, Samuel Klein

**Affiliations:** ^1^ Center for Human Nutrition Division of Geriatrics and Nutritional Science Department of Medicine Washington University School of Medicine St. Louis Missouri

**Keywords:** Muscle injury, percutaneous biopsy, ER stress, inflammation, metabolism

## Abstract

Endoplasmic reticulum (ER) stress is likely involved in the pathogenesis of metabolic dysfunction in people with obesity and diabetes. Although tissue biopsy is often used to evaluate the presence and severity of ER stress, it is not known whether acute tissue injury‐induced by percutaneous muscle biopsy causes ER stress and its potential downstream effects on markers of inflammation and metabolic function. In this study, we tested the hypothesis that percutaneous biopsy‐induced tissue injury causes ER stress and alters inflammatory and metabolic pathways in skeletal muscle. Vastus lateralis muscle tissue was obtained by percutaneous biopsy at 0600 h and 12 h later from either the contralateral leg (Group 1, *n* = 6) or at the same site as the initial biopsy (Group 2, *n* = 6) in women who were overweight. Muscle gene expression of selected markers of ER stress, inflammation, and regulators of glucose and lipid metabolism were determined. Compared with Group 1, muscle gene expression in the second biopsy sample obtained in Group 2 demonstrated marked increases in markers of ER stress (*GRP78, XBP1, ATF6*) and inflammation (*IL6, TNF*), and alterations in metabolic regulators (decreased expression of *GLUT4* and *PPARGC1A* and increased expression of *FASN*). Our results suggest that acute tissue injury induced by percutaneous muscle biopsy causes an integrated local response that involves an induction of ER stress and alterations in markers of inflammation and regulators of glucose and lipid metabolism.

## Introduction

The endoplasmic reticulum (ER) is an intracellular organelle that coordinates the synthesis, folding, and trafficking of proteins for intracellular, transmembrane and extracellular destinations. Unfolded or misfolded proteins accumulate inside the ER lumen when the normal balance between the ER protein load and its capacity to handle the load is disturbed. This situation initiates an unfolded protein response (UPR) to prevent cell damage by stopping protein translation, increasing the degradation of abnormal proteins and increasing the transcription of protein‐folding chaperones (Ron and Walter 2007; Walter and Ron 2011). However, ER stress and ER stress‐induced apoptosis can be harmful and can cause local and systemic inflammation and metabolic abnormalities (Gregor et al. 2009; Hotamisligil 2006; Hummasti and Hotamisligil 2010; Ozcan et al. 2004; Zhang and Kaufman 2008). Data obtained from studies conducted in rodents and cultured myotubes have demonstrated that ER stress impairs glucose uptake and causes muscle insulin resistance (Koh et al. 2013; Peng et al. 2011; Raciti et al. 2010; Salvado et al. 2013). In addition, ER stress in muscle is associated with obesity, insulin resistance, and type 2 diabetes in both rodents and people (Alibegovic et al. 2010; Deldicque et al. 2010; Koh et al. 2013). These findings underscore the potential importance of skeletal muscle ER stress in the pathogenesis of metabolic dysfunction.

The presence and severity of ER stress in muscle tissue can be determined by evaluating the activation of the ER transmembrane stress sensors and the three major branches of the UPR (Ron and Walter 2007; Walter and Ron 2011). In studies conducted in people, skeletal muscle samples are often obtained by percutaneous biopsy. Data obtained from previous studies have demonstrated that muscle injury causes a local inflammatory response (Aronson et al. 1998; Friedmann‐Bette et al. 2012; Guerra et al. 2011; Malm et al. 2000; Van Thienen et al. 2014) and increased production of reactive oxygen species (Furuno and Goldberg 1986; Kozakowska et al. 2015), which can increase proteolysis and protein misfolding. Although this metabolic milieu should involve ER stress, it is not known whether acute tissue injury induced by percutaneous biopsy causes ER stress in skeletal muscle in people, and whether biopsy‐induced alterations in the UPR are associated with alterations in muscle markers of inflammation and metabolic function. This issue has important implications regarding the effect of ER stress on muscle metabolic function and the use of repeated muscle biopsies when evaluating the effect of an acute intervention on ER stress.

The purpose of this study was to investigate the effects of acute tissue injury induced by percutaneous muscle biopsy on markers of ER stress, inflammation, and metabolic function. We hypothesized that a percutaneous muscle biopsy would cause a complex adaptive response involving local ER stress, inflammation, and metabolic dysfunction. Accordingly, we evaluated skeletal muscle gene expression of selected markers of ER stress (*GRP78/BiP*,* XBP1*, and *ATF6*) inflammation (*IL6* and *TNF*), and metabolic function (*GLUT4, PPARGC1A* and *FASN*) in quadriceps muscle biopsy samples obtained at 0600 h and 12 h later at the same site as the initial biopsy or the contralateral leg in women who were overweight with normal glucose tolerance.

## Materials and Methods

### Study subjects

Twelve women who were overweight enrolled in this study as part of their participation in another study that evaluated diurnal variation in metabolic function (Yoshino et al. 2014). All potential participants completed a medical evaluation, including an oral glucose tolerance test; those with impaired fasting glucose, impaired glucose tolerance, diabetes, or >1 metabolic syndrome criterion were excluded. Written informed consent was obtained from all subjects before their participation in this study, which was approved by the Institutional Review Board of Washington University School of Medicine in St. Louis, MO. This study was registered as trial number NCT02011581 in Clinical Trials.gov.

## Experimental procedures

Body composition was assessed by using dual energy X‐ray absorptiometry (Yoshino et al. 2014). Subjects were admitted to the inpatient Clinical Research Unit (CRU) at Washington University School of Medicine in the evening before the study. Subjects consumed a standard dinner at 1800 h. The following morning at 0600 h, after subjects fasted for 11 h overnight, about 150 mg of skeletal muscle (vastus lateralis) was obtained by percutaneous biopsy while the participant was lying supine in bed. This procedure involves cleaning and prepping the biopsy site and anesthetizing the skin, subcutaneous tissue, and muscle fascia with 2% lidocaine. After making a small (0.5 cm) skin incision, muscle samples were obtained by using a 5 mm Wilde Ethmoid Rongeur through the incision into the muscle bed. Muscle samples were gently rinsed with ice‐cold saline, immediately frozen in liquid nitrogen and stored at −80°C for subsequent analyses. Pressure was applied to the biopsy site for several minutes after the procedure to achieve hemostasis, before closing the incision with Steristrips, covering the site with Tegaderm and wrapping the site with an Ace bandage. A second muscle biopsy was obtained 12 h later at 1800 h from either the contralateral leg (Group 1, *n* = 6) or the same leg through the same skin incision (Group 2, *n* = 6). Subjects were given a meal at 0700 h and an identical meal at 1230 h (5.5 h before the second biopsy) and remained in bed to avoid the influence of physical activity on our outcome measures.

### Sample analyses

Total RNA was isolated from frozen skeletal muscle samples using Trizol reagent (#15596018; Invitrogen, Carlsbad, CA). RNA was quantified spectrophotometrically (NanoDrop 1000, Thermo Scientific, Waltham, MA) and reverse transcribed, using the High‐capacity cDNA Reverse Transcription Kit (#4368813; Invitrogen). Real‐time PCR was performed to assess gene expression of selected markers of markers of ER stress (glucose‐related protein 78 [*GRP78* aka *BiP*], X‐box‐binding protein 1 [*XBP1*], activating transcription factor 6 [*ATF6*]) and inflammation (interleukin 6 [*IL6*], tumor necrosis factor [*TNF*]), and selected metabolic regulators (glucose transporter 4 [*GLUT4*], peroxisome proliferator‐activated receptor gamma coactivator 1‐alpha [*PPARGC1A*], fatty acid synthase [*FASN*]). Gene expression was determined by using an ABI 7500 real‐time PCR system (Invitrogen) with SYBR Green or TaqMan (Invitrogen) as we previously described (Stromsdorfer et al. 2016; Yamaguchi et al. 2017; Yoshino et al. 2014, 2012). We purchased predesigned TaqMan probes from Invitrogen. Sequences of the primers used for this study are provided in Table 1. The expression of each gene was determined by normalizing the Ct (cycle threshold) value of each sample to the housekeeping control gene, glyceraldehyde‐3‐phosphate dehydrogenase (*GAPDH*).

**Table 1:** Sequence of primers used for RT‐PCR

Gene	Accession No.	Forward (F) and reverse (R) primer	
*GRP78*	NM_005347	F:	5′‐ CATCACGCCGTCCTATGTCG ‐3′
		R:	5′‐ GTCAAAGACCGTGTTCTCG ‐3′
*XBP1*	NM_005080	F:	5′‐ CAGACTACGTGCACCTCTGC ‐3′
		R:	5′‐ CTTCTGGGTAGACCTCTGGG ‐3′
*ATF6*	NM_007348	F:	5′‐ TCCTCGGTCAGTGGACTCTTA‐3′
		R:	5′‐ CTTGGGCTGAATTGAAGGTTTTG‐3′
*IL6*	NM_001318095	F:	5′‐ CCTGAACCTTCCAAAGATGG ‐3′
		R:	5′‐ TGGCTTGTTCCTCACTACTCTC ‐3′
*TNF*	NM_000594	F:	5′‐ TGCCTGCTGCACTTTGGAGTGA ‐3′
		R:	5′‐ TGAGGGTTTGCTACAACATGGGCT ‐3′
*GLUT4*	NM_001042	F:	5′‐ TGGGCGGCATGATTTCCTC ‐3′
		R:	5′‐ GCCAGGACATTGTTGACCAG ‐3′
*PPARGC1A*	NM_013261	F:	5′‐ TCTGAGTCTGTATGGAGTGACAT ‐3′
		R:	5′‐ CCAAGTCGTTCACATCTAGTTCA ‐3′
*FASN*	NM_004104	F:	5′‐ TGGAAGTCACCTATGAAGCCA ‐3′
		R:	5′‐ ACGAGTGTCTCGGGGTCTC ‐3′
*GAPDH*	NM_002046	F:	5′‐ TTGCCATCAATGACCCCTTCA ‐3′
		R:	5′‐ CGCCCCACTTGATTTTGGA ‐3′

### Statistical analyses

All gene expression datasets were log‐transformed for statistical analyses. The effects of the initial biopsy (at 0600 h) on gene expression at the second biopsy (at 1800 h) were determined by repeated‐measures analysis of variance (ANOVA) with time as the within‐subjects factor (0600 h vs. 1800 h) and the group as the between‐subjects factor (Group 1 vs. Group 2). When significant group x time interactions were detected, Tukey's post hoc procedure was performed to locate significant mean differences. Differences between groups at baseline were determined by a two‐tailed unpaired Student's *t* test. A *P *< 0.05 was considered statistically significant.

## Results

### Study subject characteristics

The baseline characteristics of the study subjects in Group 1 (contralateral leg second biopsy) and Group 2 (same site second biopsy) are shown in Table 2. All subjects were overweight without significant differences in age, body composition or metabolic variables between groups.

**Table 2:** Subject characteristics

	Group 1 (*n* = 6)	Group 2 (*n* = 6)
Age (years)	46.7 ± 7.4	40.0 ± 6.2
Body mass index (kg/m^2^)	28.3 ± 1.6	28.3 ± 1.3
Fat‐free mass (kg)	49.3 ± 6.4	46.1 ± 6.1
Total body fat (%)	41.1 ± 4.9	40.8 ± 5.3
Glucose (mg/dL)	95.8 ± 7.2	96.0 ± 8.2
Insulin (mU/L)	7.2 ± 3.0	7.0 ± 3.2
Triglyceride (mg/dL)	91 ± 37	57 ± 18
HDL‐cholesterol (mg/dL)	57 ± 5	69 ± 28
LDL‐cholesterol (mg/dL)	124 ± 38	84 ± 30

Values are means ± SD.

HDL, high‐density lipoprotein; LDL, low‐density lipoprotein.

### Acute tissue injury induced by percutaneous biopsy markedly increases markers of ER stress inflammation and alters metabolic regulators

**Figure 1:**
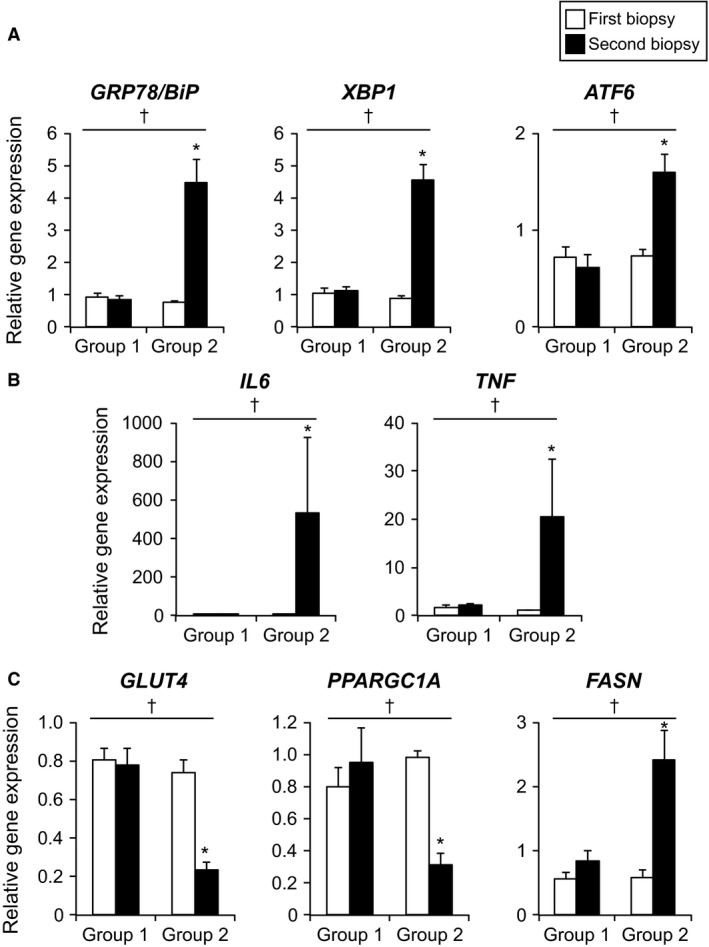
Skeletal muscle gene expression of selected markers of ER stress and inflammation, and selected metabolic regulators. Gene expression of selected markers of ER stress (*GRP78/BiP*,*XBP1*, and *ATF6*) (A), inflammation (*IL6* and *TNF*) (B), and selected metabolic regulators (*GLUT4, PPARGC1A* and *FASN*) (C) in vastus lateralis muscle samples in the first biopsy obtained at 0600 h (white bars) and the second biopsy obtained at 1800 h (black bars) from the contralateral leg (Group 1, *n* = 6) or the same site (Group 2, *n* = 6). The expression of genes of interest was normalized to *GAPDH* expression levels. Data were log‐transformed for statistical analyses and are back‐transformed for presentation. ^†^Statistically significant group x time interaction (repeated‐measures ANOVA,* P* < 0.01). *Value significantly different from all other values (Tukey's post hoc. *P* < 0.05). Data are presented as means ± SEM. *GRP78* (aka *BiP*), Glucose‐related protein 78; *XBP1,* X‐box‐binding protein 1; *ATF6,* activating transcription factor 6; *IL6*, interleukin 6; *TNF*, tumor necrosis factor; *GLUT4,* glucose transporter 4; *PPARGC1A,* peroxisome proliferator‐activated receptor gamma coactivator 1‐alpha; *FASN,* fatty acid synthase; ER, Endoplasmic reticulum.

Muscle gene expression of markers of ER stress (*GRP78/BiP*,* XBP1*, and *ATF6*) in the initial biopsy sample obtained at 0600 h was not different between Groups 1 and 2 (Figure 1A). However, compared with the initial muscle sample, gene expression of *GRP78/BiP, XBP1* and *ATF6* markedly increased in the second biopsy obtained at 1800 h in Group 2 subjects (same site second biopsy) but were not different in muscle samples obtained in Group 1 subjects (Figure 1A). Muscle gene expression of selected inflammatory markers (*IL6* and *TNF*) and metabolic regulators (*GLUT4*,* PPARGC1A*, and *FASN*) in the initial biopsy sample obtained at 0600 h were not different between Groups 1 and 2 (Figures 1B and C). However, gene expression of *IL6* and *TNF* markedly increased in the second muscle sample obtained at the same site at 1800 h (Group 2), but did not change in the second muscle sample obtained in contralateral leg (Group 1) (Figure 1B). Compared with Group 1 subjects, muscle gene expression of *GLUT4* and *PPARGC1A* markedly decreased and gene expression of *FASN* markedly increased in the second biopsy sample obtained at 1800 h in Group 2 subjects (Figure 1C).

## Discussion

In this study, we investigated the effects of acute tissue injury induced by percutaneous skeletal muscle biopsy on selected markers of ER stress, inflammation, and metabolic regulators in women who were overweight with normal glucose tolerance. Our results demonstrate that percutaneous muscle biopsy causes local ER stress, increases markers of inflammation (expression of *IL6* and *TNF)*, and alters the transcription of selected proteins involved in regulating glucose and lipid metabolism (*GLUT4*,* PPARGC1A*, and *FASN*) in tissue samples obtained 12 h after the initial biopsy. These results demonstrate that the ER is involved in a complex multi‐pathway adaptive response to acute muscle tissue injury.

Changes in the expression of ER stress markers occurred in conjunction with alterations in muscle expression of selected proteins involved in inflammation and in regulating glucose and lipid metabolism. The increase in the local expression of inflammatory markers 12 h after the initial biopsy procedure is consistent with the results from previous studies (Aronson et al. 1998; Friedmann‐Bette et al. 2012; Guerra et al. 2011; Malm et al. 2000; Van Thienen et al. 2014). Muscle biopsy also caused a marked decrease in the expression of *GLUT4* (an insulin‐sensitive glucose transporter (Henriksen et al. 1990)) and *PPARGC1A* (a major regulator of GLUT4 (Michael et al. 2001) and mitochondrial biogenesis), which has been observed previously (Friedmann‐Bette et al. 2012; Van Thienen et al. 2014), and increased the expression of *FASN* (a lipogenic enzyme that is also involved in regulating muscle insulin sensitivity (Funai et al. 2013)). Moreover, data obtained from previous studies have demonstrated that ER stress itself increases the expression of *IL6* and *TNF* in various cell types (Li et al. 2005; Zhang and Kaufman 2008), and causes a decrease in the expression of *GLUT4* and *PPARGC1A* in human myotubes (Raciti et al. 2010) and an increase in de novo lipogenesis in mouse models (Lee et al. 2008; Rutkowski et al. 2008). Taken together, these results suggest that ER stress, inflammation, and alterations in metabolic function are components of an integrated acute response to muscle injury induced by percutaneous biopsy.

Our finding that percutaneous biopsy causes local ER stress in muscle tissue is similar to the effect of open excisional biopsy on ER stress in subcutaneous adipose tissue reported previously (Boden et al. 2010). These findings suggest that tissue injury induces a universal metabolic response that involves ER stress, inflammation, and alterations in the regulation of metabolic pathways. We did not determine whether the induction of local ER stress in muscle influenced muscle tissue or systemic metabolic function. However, it is unlikely that such a small tissue injury would have widespread metabolic effects. In fact, it has previously been shown that biopsy‐induced ER stress in subcutaneous adipose tissue did not affect whole‐body insulin sensitivity, assessed by the hyperinsulinemic‐euglycemic clamp procedure (Boden et al. 2010). Nonetheless, it is possible that greater muscle damage and ER stress would have systemic effects on metabolic function, which could help explain the insulin resistance associated with muscle injury induced by surgery (Thorell et al. 1994) and trauma (Cree and Wolfe 2008; Jeschke et al. 2012).

Our study has several important limitations. First, we did not assess protein content, protein modification or functional measures of metabolic activity because of limited tissue availability, so we cannot exclude the possibility that the observed transcriptional changes were accompanied by changes in protein levels of inflammatory cytokines or more direct measures of muscle metabolic function. In addition, muscle biopsies were obtained by using a 5‐mm Wilde Ethmoid Rongeur, so our results might not be representative of the effects of percutaneous biopsies obtained by using other instruments, such as a Bergström needle.

In conclusion, the results from this study demonstrate that acute tissue injury induced by percutaneous muscle biopsy causes a complex local response that involves ER stress, inflammation, and alterations in the expression of selected regulators of glucose and lipid metabolism. Additional studies are needed to determine the precise mechanisms responsible for sensing tissue injury and integrating these different response pathways to repair cell damage and maintain cellular homeostasis.

## Conflict of Interest

The authors report no conflicts of interest in this work.
